# Supplementary material for: OSBPL11 is an African-specific locus associated with 25-hydroxyvitamin D concentrations and cardiometabolic health
Source: medRxiv. 2025 May 31:2025.05.27.25328359. Preprint. [Version 1] doi: 10.1101/2025.05.27.25328359 (PMC12148284; doi:10.1101/2025.05.27.25328359)
Supplement: Supplement 2 [file NIHPP2025.05.27.25328359v1-supplement-2.pdf]

Supplementary Appendix

Supplementary Table 1. Characteristics of study participants

	Discovery cohorts					Replication cohorts				
	**Kenyans**	**Ugandans**	**Burkinabe**	**Gambians**	**South Africans**	**African Brazilian (SCAALA)**	**African American Adults from JHS**	**Multi-Ethnic Study of Atherosclerosis (MESA)**	**African ancestry UK (UKB)**	**African American (All of US)**
No. of participants[§]	962	1288	329	484	607	753	5306	1677	8151	7400
Median 25(OH)D nmol/L (IQR)[*]	78.1 (64.0, 96.5)	78.6 (65.0, 94.6)	78.6 (65.1, 94.5)	72.4 (59.6, 84.9)	72.7 (58.7, 88.8)	27.4 (21.3, 33.1)	32.4 (22.5, 46.2)	17.4 (12.1, 24.2)	31.0 (22.1, 42.9)	26.0 (17.0, 37.0)
Median (IQR) age[†]	21.6 (15.2, 39.3) m	24.0 (23.9, 35.9) m	24.1 (23.9, 35.9) m	46.6 (34.6, 58.4) m	12.0 (11.9, 12.2) m	7.0 (6.0, 9.0) y	55.0 (45.0, 64.0) y	62.0 (53.0, 70.0)	50.0 (45.0, 58.0) y	61.0 (50.0, 69.0) y
Sex: females	473/962 (49.2%)	638/1288 (49.5%)	161/329 (48.9%)	218/484 (45.0%)	291/607 (47.9%)	361/750 (48.1%)	3367/5306 (63.1%)	906 (54.0%)	3393/8151 (41.6%)	6458/8935 (72.28%)
Season[#]										
1st season	48/962 (5.0%)	302/1284 (23.4%)	72/329 (21.9%)	-	238/607 (39.2%)	179 (24.2%)	1137/5306 (21.4%)	409/1677(24.4%)	1719/8151 (21.1%)	2255/8935 (25.2%)
2nd season	357/962 (37.1%)	310/1284 (24.1%)	123/329 (37.4%)	-	25/607 (4.1%)	-	1189/5306 (22.4%)	565/1677(33.6%)	2197/8151 (27.0%)	1889/8935 (21.1%)
3rd season	528/962 (54.9%)	318/1284 (24.6%)	129/329 (39.2%)	536/484 (85.4%)	228/607 (37.6%)	-	1447/5306 (27.3%)	339/1677(20.2%)	1565/8151 (19.2%)	2220/8935 (24.9%)
4th season	29/962 (3.0%)	354/1284 (27.5%)	5/329 (1.5%)	92/484 (14.7%)	116/607 (19.1%)	562 (75.8%)	1533/5306 (28.9%)	364/1677(21.7%)	2670/8151 (32.7%)	2571/8935 (28.8%)
BMI (IQR)	14.9 (13.7, 16.)	15.7 (14.7, 16.4)	15.6 (14.7, 16.4)	14.6 (13.8, 15.4)	n/a	15.6 (14.3, 16.3)	30.5 (26.9, 35.4)	29.4 (26.1, 33.5)	28.7 (25.7, 32.3)	32.1 (27.1, 38.4)
Malaria[¶]	189/857 (22.1%)	88/1268 (6.9%)	64/303 (21.1%)	65/484 (10.4%)	[¶]No malaria	[¶]No malaria	[¶]No malaria	[¶]No malaria	[¶]No malaria	[¶]No malaria
Inflammation[‡]	256/962 (27.0%)	304/1273 (23.9%)	109/329 (33.9%)	85/484 (14%)	94/607 (15.5%)	230/751(30.5%)	1242/4791(25.9%)[§§]	445/1677 (26.6%)	1088/8151 (13%)	n/a
Supplementation[††]	n/a[††]	n/a[††]	n/a[††]	n/a[††]	n/a[††]	n/a[††]	1648/5306 (34.4%)	n/a	490/7611 (6.4%)	5640/8935 (63.1%)

Abbreviations; IQR, interquartile range; n/a, not available; 25(OH)D, 25-hydroxyvitamin D. [§]The number of participants in each study cohorts included in the GWAS, based on availability of genotype and phenotype data. [¶]Malaria was defined as the presence of *P. falciparum* parasites on blood film. [‡]Inflammation was defined as CRP > 5 mg/L or ACT > 0.6 g/L. ACT, but not CRP, was available for The Gambia. [†]Age is presented in months for discovery cohorts and years for replication cohorts. [#]1st season corresponds to summer/short rains/dry season; 2nd season as autumn/dry season; 3rd season as winter/long rains season; 4th season as spring or dry season. Seasons in discovery cohorts were based on 3 monthly intervals as follows 1st season, December to February; 2nd season, March to May; 3rd season, June to August; 4th season, September to November. In South Africa these seasons correspond to summer, autumn, winter, and spring, respectively, in Uganda and Kenya there are two rainy and two dry seasons and in Burkina Faso and The Gambia there is a single rainy and dry season. For MESA study, seasons were defined as follows;1st season from January to March; 2nd, April to June; 3rd, July to September; 4th, October to December. However, the timing of the rains is often unpredictable and may vary from these times. [§§]HsCRP was measured in African American cohorts, which was converted to values equivalent to CRP as previously (Arthroplasty 2014). [††]Vitamin D supplementation is very low/non-existent in the African children.

Supplementary Table 2. Fine-mapping posterior probabilities of *k* causal variants at independent signal loci that reached genome-wide significance in the discovery GWAS

Locus	GWAS index variant	Chromosome: position	K (no. of causal variants) *	Posterior probability
GC	rs1352846	4:72617775	**26**	**1**
			27	2.96×10^{-10}
CYP2R1/PDE3B/PSMA1	rs12290926	11:14695046	**24**	**0.54**
			25	0.30
			21	0.056
			22	0.040
			23	0.046
			26	0.021
DHCR7/NADSYN1	rs7950991	11:71158612	**11**	**1**
			10	2.96×10^{-106}
OSBPL11	rs2979356	3:125367492	**8**	**1**

*K (causal variants) 0 to 100 were analyzed. However, only *ks* with non-zero posterior probabilities are presented in this table. The most probable no of causal variants (k) is highlighted in bold. The *k* variants listed are predicted as possible credible sets and are not definitively causal.

medRxiv preprint doi: https://doi.org/10.1101/2025.05.27.25328359; this version posted May 31, 2025. The copyright holder for this preprint (which was not certified by peer review) is the author/funder, who has granted medRxiv a license to display the preprint in perpetuity.
It is made available under a CC-BY 4.0 International license .

Supplementary Table 3. Effect of independent African genome-wide significant variants in continental and diaspora African ancestry cohorts and Europeans in the UK Biobank

GENE	*GC*	*CYP2R1/PDE3B/PSMA1*	*DHCR7/NADSYN1*
**VARIANT DETAILS**			
Lead variant	rs1352846	rs12290926	rs7950991
Chromosome: position	4:72617775	11:14695046	11:71158612
Effect/ref allele	A/G	A/G	G/T
Functional effect	intronic	intronic	intronic
**CONTINENTAL AFRICAN ANCESTRY COHORTS**			
**West Africans meta-analysis**			
Allele Freq.	0.039	0.14	0.15
Effect (Beta)	-0.19	-0.25	0.19
SE	0.11	0.063	0.059
P value	0.084	5.45×10^{-05}	0.0012
**East Africans meta-analysis**			
Allele Freq.	0.10	0.13	0.16
Effect (Beta)	-0.32	-0.17	0.25
SE	0.047	0.041	0.038
P value	3.04×10^{-11}	3.41×10^{-05}	5.06×10^{-11}
**South Africans**			
Allele Freq.	0.066	0.19	0.200
Effect (Beta)	-0.23	-0.21	0.16
SE	0.11	0.073	0.072
P value	0.040	0.0037	0.029
**DIASPORA AFRICAN ANCESTRY COHORTS**			
**SCAALA (Brazil)**			
Allele Freq.	0.163	0.30	0.10
Effect (Beta)	-0.075	-0.12	0.17
SE	0.069	0.056	0.087
P value	0.28	0.039	0.050
**JHS (USA)**			
Allele Freq.	0.10	0.15	0.17
Effect (Beta)	-0.12	-0.02	0.10
SE	0.038	0.031	0.030
P value	0.0016	0.56	3.97×10^{-4}
**MESA (USA)**			
Allele Freq.	0.116	0.172	0.155
Effect (Beta)	-0.218	0.0453	0.0188
SE	0.0610	0.0523	0.0530
P value	0.00036	0.39	0.72
**All of Us (USA)**			
Allele Freq.	0.10	0.16	0.17
Effect (Beta)	-0.124	-0.0593	0.0717
SE	0.026	0.0218	0.0208
P value	2.18×10^{-06}	0.0065	0.00056
**UK Biobank (UK)**			
Allele Freq.	0.09	0.15	0.16
Effect (Beta)	-0.19	-0.02	0.07
SE	0.03	0.02	0.02
P value	6.66×10^{-12}	0.48	6.40×10^{-4}
**EUROPEAN ANCESTRY COHORT**			
**UK Biobank (UK)**			
Allele Freq.	0.291	0.41	n/a*
Effect (Beta)	-0.187	-0.068	n/a*
SE	0.002	0.002	n/a*
P value	2.90×10^{-1689}	3.53×10^{-266}	n/a*

Abbreviation: ref. reference; SE standard error; freq. frequency; n/a not available. UKB European summary data was obtained from Minousaki et al[3]. The discovery association analyses were adjusted for age, sex and season, and incorporated GRM to account for relatedness. The rs7950991 variant was missing in European GWAS, it is rare in Europeans (MAF<0.001, dbSNP). However, rs12284909, which is in high linkage disequilibrium with rs7950991 in our discovery cohorts (r^2=0.98), was genome-wide significant in Europeans (effect/other allele; A/G, A allele frequency=0.006, beta = -0.09, P=7.6×10^{-12}).

Supplementary Table 4. Replication of genome-wide significant variants in UK Biobank European-ancestry GWAS on 25(OH)D concentrations in a meta-analysis of continental African cohorts

				UKB European population GWAS				Continental African populations GWAS			
**Gene**	**Variant**	**Chromosome: position**	**Effect/ref allele**	**Allele Freq.**	**Effect (Beta)**	**SE**	***P* value**	**Allele Freq.**	**Effect (Beta)**	**SE**	***P* value**
CYP2R1	rs201501563	11:14882470	T/C	0.065	-0.035	0.004	1.96×10^{-18}	0.084	-0.215	0.040	6.01×10^{-08}
CYP2R1	rs10832289	11:14669496	T/A	0.41	-0.086	0.002	2.84×10^{-266}	0.088	-0.204	0.039	1.31×10^{-07}
GC	rs11723621	4:72615362	G/A	0.29	-0.156	0.003	2.90×10^{-1689}	0.069	-0.182	0.043	2.61×10^{-05}
SEC23A	rs8018720	14:39556185	C/G	0.82	-0.032	0.003	4.10×10^{-36}	0.86	-0.091	0.031	0.003
RP13-379L11.3	rs2585442	20:52737123	G/C	0.24	0.023	0.002	3.96×10^{-23}	0.069	0.114	0.042	0.007
COG5	rs1858889	7:107117447	C/A	0.50	0.013	0.002	3.03×10^{-11}	0.21	0.068	0.026	0.010
HAL	rs10859995	12:96375682	C/T	0.59	-0.041	0.002	3.03×10^{-91}	0.34	-0.045	0.023	0.050
STAP2	rs57631352	19:4338173	G/A	0.30	-0.013	0.002	1.50×10^{-09}	0.31	0.040	0.023	0.089
MAT1A	rs10887718	10:82042624	T/C	0.53	-0.013	0.002	1.18×10^{-10}	0.43	0.036	0.022	0.100
GC	rs222026	4:72643760	T/A	0.88	-0.051	0.004	1.09×10^{-40}	0.34	-0.036	0.023	0.117
LDLR	rs73015021	19:11192915	G/A	0.12	0.022	0.003	6.29×10^{-14}	0.37	0.034	0.023	0.138
RP11-120M18.2	rs2909218	17:66464546	T/C	0.80	0.017	0.002	2.82×10^{-12}	0.75	-0.037	0.025	0.138
APOC1	rs1065853	19:45413233	T/G	0.081	0.028	0.004	2.24×10^{-14}	0.13	0.047	0.032	0.140
RP13-379L11.3	rs6123359	20:52714706	G/A	0.10	0.024	0.003	7.48×10^{-14}	0.090	0.056	0.039	0.145
TMEM151A	rs523583	11:66070146	C/A	0.47	0.013	0.002	6.60×10^{-12}	0.12	-0.047	0.033	0.159
GCKR	rs11127048	2:27752463	A/G	0.62	0.018	0.002	6.72×10^{-19}	0.76	0.036	0.025	0.159
RP11-21L23.4	rs1149605	11:76485216	C/T	0.17	0.020	0.003	3.36×10^{-15}	0.11	0.048	0.034	0.161
RP4-657M3.2	rs7519574	1:34726552	A/G	0.18	0.017	0.003	4.03×10^{-11}	0.13	-0.045	0.033	0.170
ABO	rs532436	9:136149830	A/G	0.19	-0.015	0.003	1.94×10^{-09}	0.11	-0.045	0.035	0.195
DSG1	rs8091117	18:28919794	A/C	0.064	-0.024	0.004	9.48×10^{-10}	0.43	0.030	0.023	0.202
KLK10	rs10426	19:51517798	A/G	0.21	0.025	0.002	1.59×10^{-26}	0.080	0.050	0.040	0.220
ZPR1	rs964184	11:116648917	C/G	0.87	0.040	0.003	1.30×10^{-43}	0.79	0.031	0.027	0.247
HSD17B11	rs58073039	4:88287363	G/A	0.30	-0.013	0.002	2.84×10^{-10}	0.57	-0.022	0.022	0.319
SCUBE1	rs960596	22:41393520	T/C	0.34	0.012	0.002	2.43×10^{-09}	0.022	-0.074	0.077	0.336
PADI1	rs3750296	1:17559656	C/G	0.34	-0.021	0.002	3.04×10^{-24}	0.44	-0.020	0.022	0.350
EBF2	rs34726834	8:25889606	T/C	0.25	0.014	0.002	3.39×10^{-10}	0.36	-0.021	0.023	0.354
SERPINB11	rs2037511	18:61366207	A/G	0.17	0.016	0.003	8.35×10^{-10}	0.078	-0.037	0.041	0.361
CELSR2	rs7528419	1:109817192	G/A	0.22	0.019	0.002	2.43×10^{-16}	0.30	0.020	0.024	0.397
TM6SF2	rs58542926	19:19379549	T/C	0.077	0.033	0.004	2.63×10^{-19}	0.017	0.072	0.088	0.411
FLJ42102	rs12803256	11:71132868	G/A	0.78	0.087	0.003	1.64×10^{-195}	0.17	0.023	0.030	0.436
TNFAIP8	rs7718395	5:118652574	G/C	0.32	0.013	0.002	1.68×10^{-09}	0.06	-0.036	0.048	0.454
ZPR1	rs2847500	11:120114421	A/G	0.12	-0.021	0.003	1.93×10^{-12}	0.32	-0.017	0.024	0.467
TDRD15	rs12997242	2:21381177	A/G	0.44	-0.012	0.002	2.32×10^{-10}	0.19	0.020	0.028	0.468

BCAR4	rs8063706	16:11909552	T/A	0.27	0.013	0.002	4.27×10^{-09}	0.50	0.016	0.022	0.472
ARNT	rs3768013	1:150815411	A/G	0.37	-0.012	0.002	3.86×10^{-09}	0.53	-0.014	0.021	0.502
MARC_1	rs867772	1:220972343	G/A	0.68	-0.014	0.002	3.31×10^{-11}	0.94	0.029	0.045	0.523
CETP	rs1800775	16:56995236	A/C	0.49	-0.017	0.002	1.57×10^{-17}	0.62	0.012	0.022	0.582
GC	rs705117	4:72608115	T/C	0.85	0.031	0.003	1.12×10^{-27}	0.19	0.015	0.027	0.590
ZNF808	rs8103262	19:53065814	C/T	0.30	0.013	0.002	6.80×10^{-10}	0.42	0.012	0.022	0.602
DOK7	rs78649910	4:3482213	A/T	0.11	-0.018	0.003	3.41×10^{-09}	0.10	-0.017	0.037	0.639
APOC1	rs157595	19:45425460	G/A	0.62	-0.016	0.002	4.25×10^{-15}	0.86	0.015	0.033	0.648
NPAS2	rs6724965	2:101440151	G/A	0.17	-0.017	0.003	1.34×10^{-10}	0.71	0.010	0.023	0.662
HTR5BP	rs7569755	2:118648261	A/G	0.29	0.014	0.002	8.35×10^{-11}	0.41	-0.010	0.023	0.664
MED23	rs3822868	6:131934986	G/A	0.84	0.022	0.003	1.41×10^{-15}	0.023	0.033	0.076	0.667
DNAH11	rs111529171	7:21571932	C/G	0.22	-0.015	0.002	6.26×10^{-11}	0.22	-0.011	0.027	0.682
MRPL3	rs6438900	3:125148287	G/C	0.26	0.014	0.002	1.16×10^{-09}	0.50	0.007	0.022	0.738
LINC00536	rs7828742	8:116960729	G/A	0.60	-0.024	0.002	2.85×10^{-33}	0.71	-0.007	0.024	0.767
PDILT	rs77924615	16:20392332	A/G	0.19	-0.016	0.002	2.28×10^{-10}	0.049	-0.014	0.051	0.782
CPS1	rs1047891	2:211540507	A/C	0.32	-0.014	0.002	1.16×10^{-11}	0.36	-0.006	0.024	0.799
NPHS1	rs3814995	19:36342212	T/C	0.31	-0.015	0.002	1.08×10^{-12}	0.037	-0.015	0.062	0.807
RHOA	rs7650253	3:49431160	A/T	0.70	0.015	0.002	1.76×10^{-10}	0.75	-0.006	0.026	0.827
UGT2B7	rs7699711	4:69947596	T/G	0.45	-0.030	0.002	4.85×10^{-50}	0.75	-0.005	0.026	0.834
CADM2	rs1972994	3:85631142	T/A	0.65	-0.018	0.002	8.04×10^{-18}	0.78	0.006	0.027	0.836
FOXO6	rs56044892	1:41830086	T/C	0.20	0.015	0.002	3.13×10^{-10}	0.074	0.009	0.044	0.839
LIPC	rs1800588	15:58723675	T/C	0.21	-0.030	0.002	3.17×10^{-37}	0.53	-0.004	0.021	0.842
RP13-379L11.3	rs6127099	20:52731402	T/A	0.27	-0.027	0.002	2.22×10^{-32}	0.18	0.006	0.030	0.849
FDPS	rs11264360	1:155284586	A/T	0.24	0.018	0.002	1.12×10^{-15}	0.22	0.003	0.026	0.908
SLCO1B1	rs12317268	12:21352541	G/A	0.15	-0.019	0.003	9.20×10^{-12}	0.16	-0.003	0.030	0.920
TFDP2	rs6773343	3:141825598	T/C	0.72	0.013	0.002	6.28×10^{-09}	0.71	-0.002	0.023	0.928
AC007950.2	rs17765311	15:63789952	C/A	0.34	-0.015	0.002	1.18×10^{-13}	0.022	0.007	0.080	0.933
LIPC	rs261291	15:58680178	C/T	0.36	-0.023	0.002	2.46×10^{-29}	0.43	-0.002	0.022	0.934
DNAH11	rs10818769	9:125719923	G/C	0.86	-0.017	0.003	2.99×10^{-09}	0.053	-0.003	0.048	0.951
RER1	rs6698680	1:2329661	G/A	0.47	-0.012	0.002	7.47×10^{-10}	0.30	-0.001	0.024	0.971
PEAK1	rs62007299	15:77711719	A/G	0.71	-0.014	0.002	3.33×10^{-11}	0.52	-0.001	0.022	0.974
GATA4	rs804280	8:11612698	A/C	0.58	0.016	0.002	9.90×10^{-16}	0.63	-2.5×10^{-4}	0.022	0.991
LINC01004	rs1011468	7:104613791	A/G	0.47	-0.014	0.002	1.39×10^{-12}	0.85	-5.5×10^{-5}	0.029	0.998

European summary data were obtained from Minousaki et al[3]. Variants that were missing in the current GWAS or had a *P* value > 5×10^{-8} in the European GWAS were excluded. The variants have been sorted by *P* value in a meta-analysis of GWAS of continental African populations. Replication was defined as the same direction of effect and *P* value < 0.05.

Supplementary Table 5. Effect of *OSBPL11* lead variant, rs2979356, on select biomarkers of lipid metabolism and cardiovascular health in African ancestry individuals

	n	Beta	SE	P
**HDL cholesterol**				
AADM (overall)	4,295	0.0338	0.0319	0.29
AADM (West Africa)	3,561	-0.0272	0.0368	0.46
AWI-Gen (overall)	10,603	0.004	0.020	0.83
AWI-Gen (West Africa)	3,763	-0.027	0.034	0.43
UKB (African ancestry)	5,754	-0.00359	0.0240	0.055
AMP-CMDKP	128,439	-0.0039	0.0039	0.47
GLGC	90,804	0.00209	0.00595	0.73
**Total cholesterol**				
AADM (overall)	4,284	0.0154	0.0319	0.63
AADM (West Africa)	3,553	0.0188	0.0366	0.61
AWI-Gen (overall)	10,603	-0.023	0.020	0.20
AWI-Gen (West Africa)	3,763	-0.031	0.033	0.36
UKB (African ancestry)	6,212	0.0174	0.0248	0.32
AMP-CMDKP	128,439	0.0086	0.0039	0.11
GLGC	92,554	-0.00928	0.00589	0.12
**HOMA2-%S** (AADM)	2,390	-0.0140	0.047	0.77
**HOMA2-B** (AADM)	2,390	-0.0181	0.036	0.61
**TG/HDL ratio** (AWI-Gen West Africa)	23,139	0.0277	0.0227	0.23
**Fasting Glucose** (AWI-Gen West Africa)	3,591	0.024	0.022	0.57
**Fasting Insulin** (AWI-Gen West Africa)	2,129	0.007	0.021	0.14

The effect/alternative allele is T/C. HOMA2-B estimates beta cell function and insulin production in the body using fasting insulin and glucose levels. HOMA2-%S is a calculation that measures how well peripheral tissues respond to insulin and absorb glucose from the bloodstream.*The AWI-Gen Study (Africa Wits-INDEPTH Partnership for Genomic Studies) aimed to investigate the genetic and environmental factors influencing cardiometabolic diseases in sub-Saharan Africa, with participants recruited from Burkina Faso, Ghana, Kenya, Namibia, South Africa, and Tanzania[37]. AWI-Gen W. Africa included The Gambia and Ghana. The AMP-CMDKP (African American Cardiometabolic Disease Knowledge Portal) aimed to explore genetic contributors to cardiometabolic diseases, recruiting participants from diverse African ancestry populations (https://hugeamp.org/). Participants in the AADM study and the UK Biobank study are described in the methods.

Supplementary Table 6. Study cohort site, design, site, genotyping and phenotyping

	Continental African discovery cohorts					African ancestry replication cohorts				
**Study name**	**Kilifi immunology cohort (Kenya)**	**The Entebbe Mother and Baby Study (Uganda)**	**The VAC050 ME-TRAP malaria vaccine trial (Burkina Faso)**	**Malaria study (The Gambia)**	**Soweto Vaccine Response Study (South Africa)**	**Jackson Heart Study (USA)**	**Multi-Ethnic Study of Atherosclerosis (USA)**	**UK Biobank African ancestry participants (UK)**	**SCAALA (Brazil)**	**All of US Research Program (USA)**
**Study Site**	Kilifi, Kenya	Entebbe, Uganda	Banfora, Burkina Faso	West Kiang, The Gambia	Soweto, South Africa	Jackson, Mississippi, USA	USA	UK	Salvador, Brazil	USA
**Study design**	Community-based cohort	Community-based cohort	Community-based cohort	Community-based cohort	Community-based cohort	Community-based cohort	Community-based cohort	Population cohort	Community-based cohort	Population cohort
**Genotyping platform**	H3Africa chip v1	Illumina HumanOmni 2.5M-8	Illumina HumanOmni 2.5M-8	H3Africa chip v1	Illumina HumanOmni 2.5M-8	Affymetrix 6.0	Affymetrix 6.0	UK Biobank Axiom™ Array	Illumina HumanOmni 2.5M	Illumina NovaSeq 6000 whole genome sequencing
**Number of variants in chip**	2.5 million	2.5 million	2.5 million	2.5 million	2.5 million	906,600	906,600	850,000	2.5 million	Whole genome sequence
**Vitamin D assay**	CMIA (Abbott Architect, USA)	CMIA (Abbott Architect, USA)	CMIA (Abbott Architect, USA)	CMIA (Abbott Architect, USA)	CMIA (Abbott Architect, USA)	LC-MS-MS	LC-MS-MS	DiaSorin Liaison XL assay	Inhibitory enzyme immunosorbent assay (Bolton, UK)	Multiple assays

CMIA, chemiluminescent microparticle immunoassay; SCAALA, Social Change Asthma and Allergy; UKB, UK Biobank; LC-MS-MS, Liquid chromatography with tandem mass spectrometry.

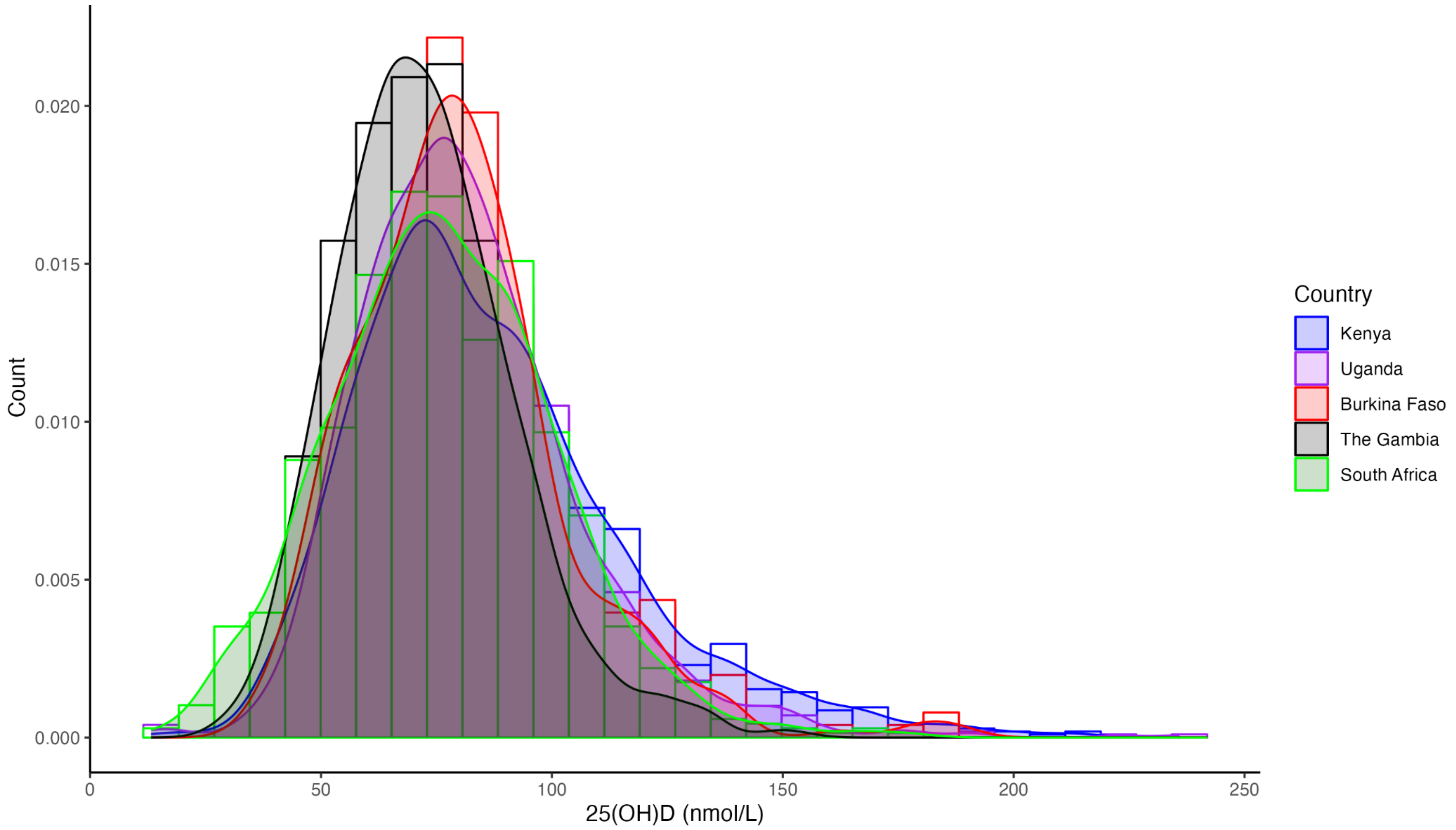

Supplementary Figure 1. Histogram with density plots showing the distribution of 25(OH)D concentrations. Shapiro-Wilk's test for normality indicated that 25(OH)D concentrations were skewed (*W*=0.95, *P* value < 0.001).

medRxiv preprint doi: https://doi.org/10.1101/2025.05.27.25328359; this version posted May 31, 2025. The copyright holder for this preprint (which was not certified by peer review) is the author/funder, who has granted medRxiv a license to display the preprint in perpetuity.
It is made available under a CC-BY 4.0 International license .

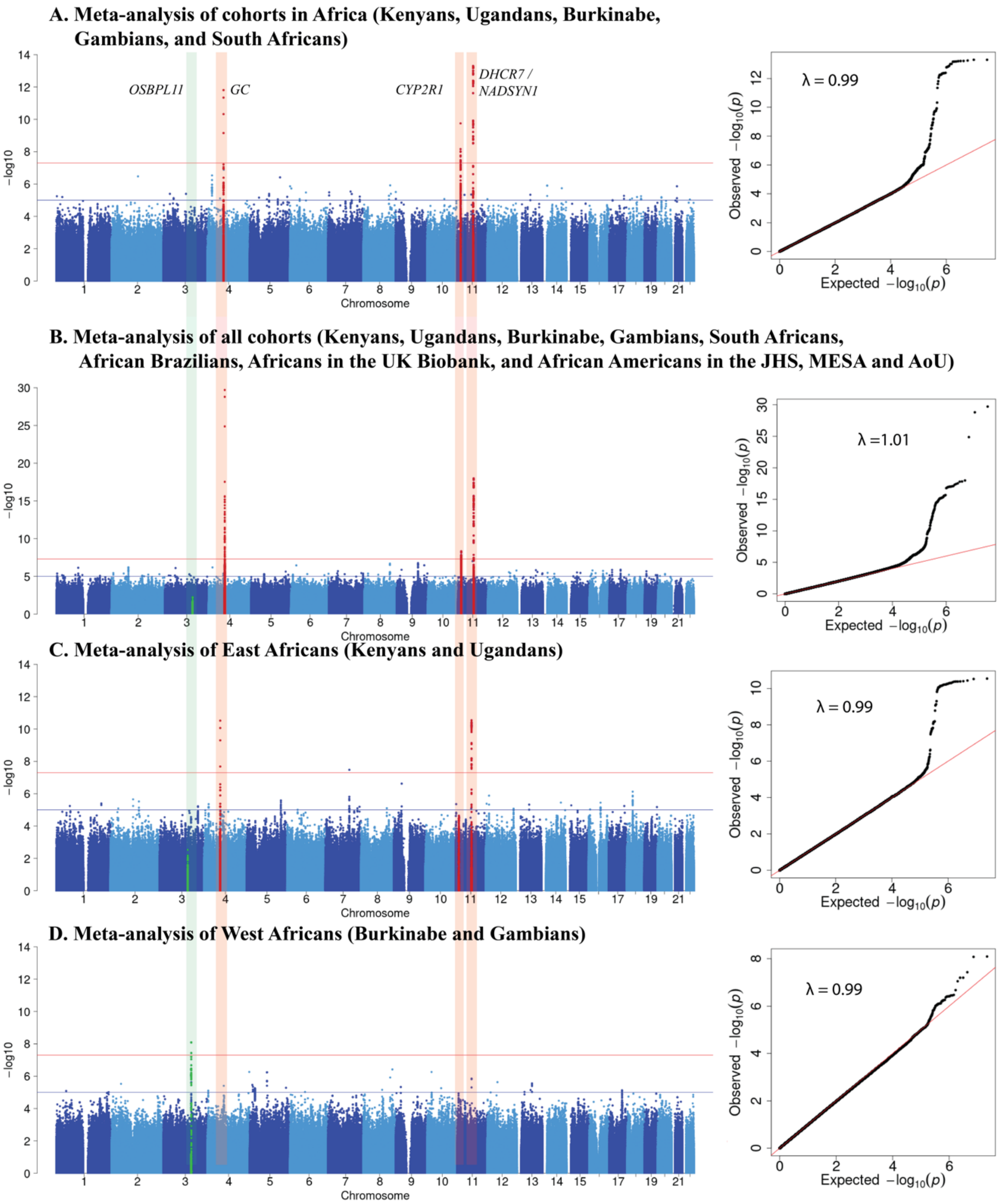

Supplementary Figure 2. Manhattan and QQ plots of GWAS of 25(OH)D concentrations meta-analysis of all cohorts in Africa (A), all discovery and replication cohorts (B), East African cohorts (C), and West African cohorts (D). Variants in known loci (*GC, CYP2R1/PDE3B* and *DHCR7/NADSYN1*) are indicated in red and novel loci (*OSBPL11*) in green.

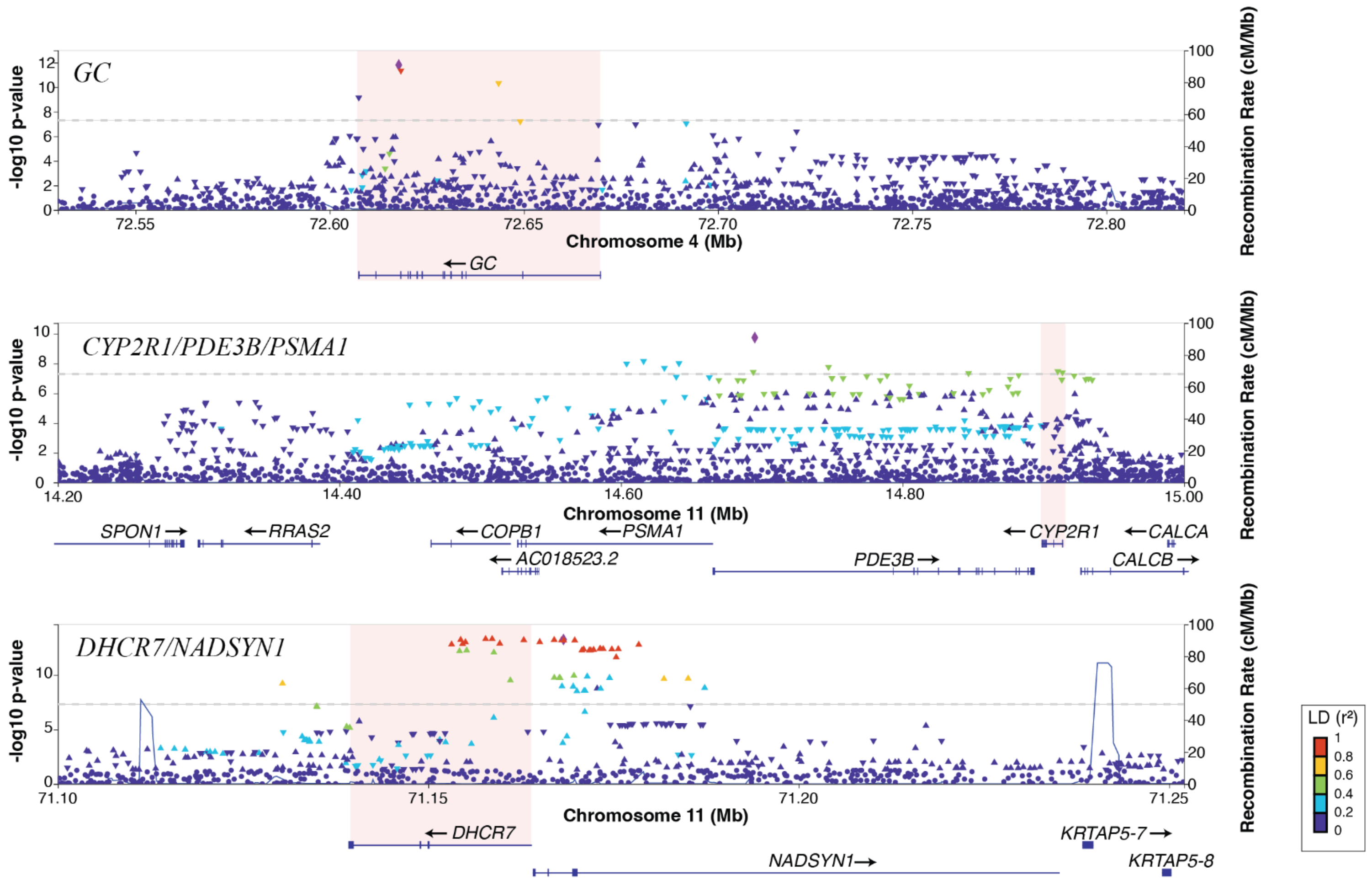

Supplementary Figure 3. Regional association plots for the *GC*, *CYP2R1/PDE3B/PSMA1* and *DHCR7/NADSYN1* loci. The top variant in the regional plots is shown with a purple diamond shape and the rest are coloured according to their LD (R^2) relative to the top variant in individuals of African ancestry in this study; colour varies between absent LD (blue) and total LD (red). The direction of the triangle of the top variant in each locus in the regional plots indicates the direction of effect on 25(OH)D concentrations.

medRxiv preprint doi: https://doi.org/10.1101/2025.05.27.25328359; this version posted May 31, 2025. The copyright holder for this preprint (which was not certified by peer review) is the author/funder, who has granted medRxiv a license to display the preprint in perpetuity.
It is made available under a CC-BY 4.0 International license .

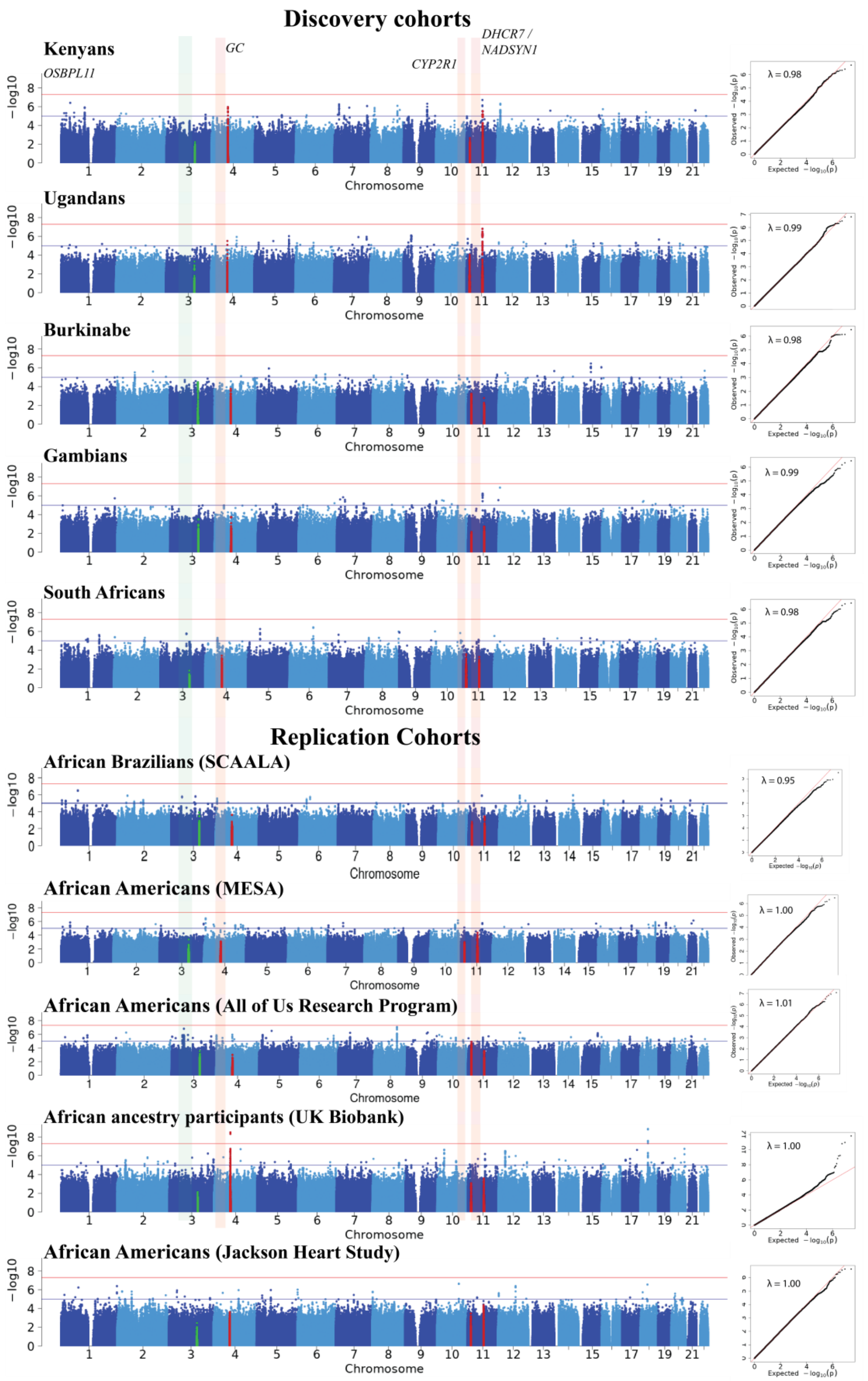

Supplementary Figure 4. Manhattan and QQ plots of GWAS of 25(OH)D concentrations by cohort. Variants in and close to known loci (*GC*, *CYP2R1/PDE3B* and *DHCR7/NADSYN1*) are shown in red and novel loci (*OSBPL11*) are shown in green.

medRxiv preprint doi: https://doi.org/10.1101/2025.05.27.25328359; this version posted May 31, 2025. The copyright holder for this preprint (which was not certified by peer review) is the author/funder, who has granted medRxiv a license to display the preprint in perpetuity.
It is made available under a CC-BY 4.0 International license .

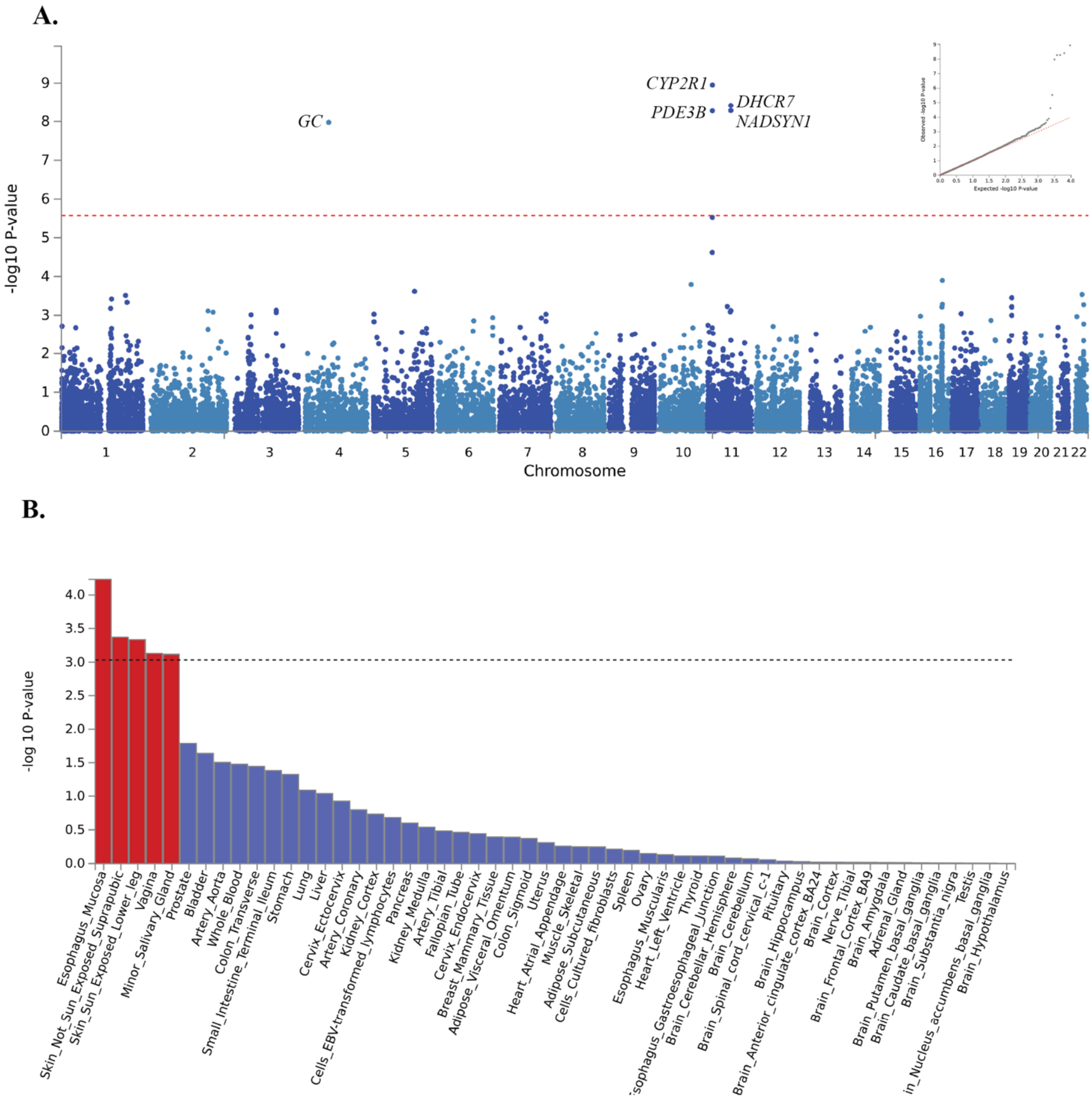

Supplementary Figure 5. Manhattan plot of MAGMA gene-based association test results and corresponding QQ plot (A) and MAGMA tissue expression analysis, where tissues that exceed the significance threshold (P = 0.05/18912 = 2.64 X 10^{-6}) are shown in red (B), Dark blue areas highlight genomic risk loci. Green lines represent eQTL associations, while orange lines depict chromatin interactions. Genes mapped by both eQTL and chromatin interactions are highlighted in red. The gene mapping was conducted using FUMA using African ancestry data (https://fuma.ctglab.nl/) with the gene-based test performed via MAGMA (v1.08) [39]. Input variants were mapped to 18,912 protein-coding genes.

medRxiv preprint doi: https://doi.org/10.1101/2025.05.27.25328359; this version posted May 31, 2025. The copyright holder for this preprint (which was not certified by peer review) is the author/funder, who has granted medRxiv a license to display the preprint in perpetuity.
It is made available under a CC-BY 4.0 International license .

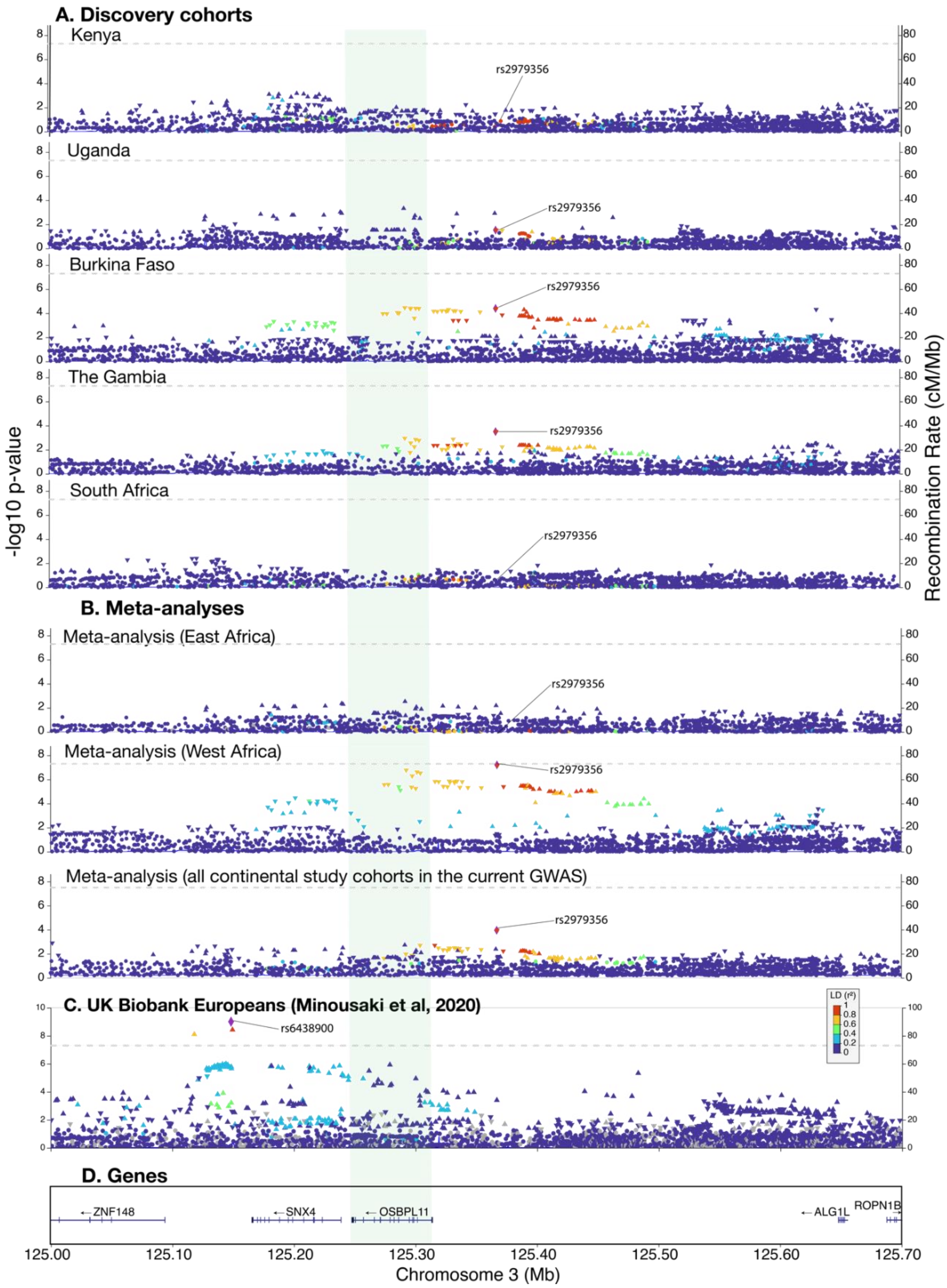

Supplementary Figure 6. Regional association plots for the *OSBPL11* locus in chromosome 3 for individual continental African cohorts (A), meta-analyses (B), UK Biobank Europeans (C), and genes in the regions (D). The direction of the triangle of the top variant indicates the direction of effect on 25(OH)D concentrations. The variants are coloured according to their LD (R^2) relative to the top variant. The reference variant (rs2979356) is illustrated in a purple diamond shape.

medRxiv preprint doi: https://doi.org/10.1101/2025.05.27.25328359; this version posted May 31, 2025. The copyright holder for this preprint (which was not certified by peer review) is the author/funder, who has granted medRxiv a license to display the preprint in perpetuity.
It is made available under a CC-BY 4.0 International license .

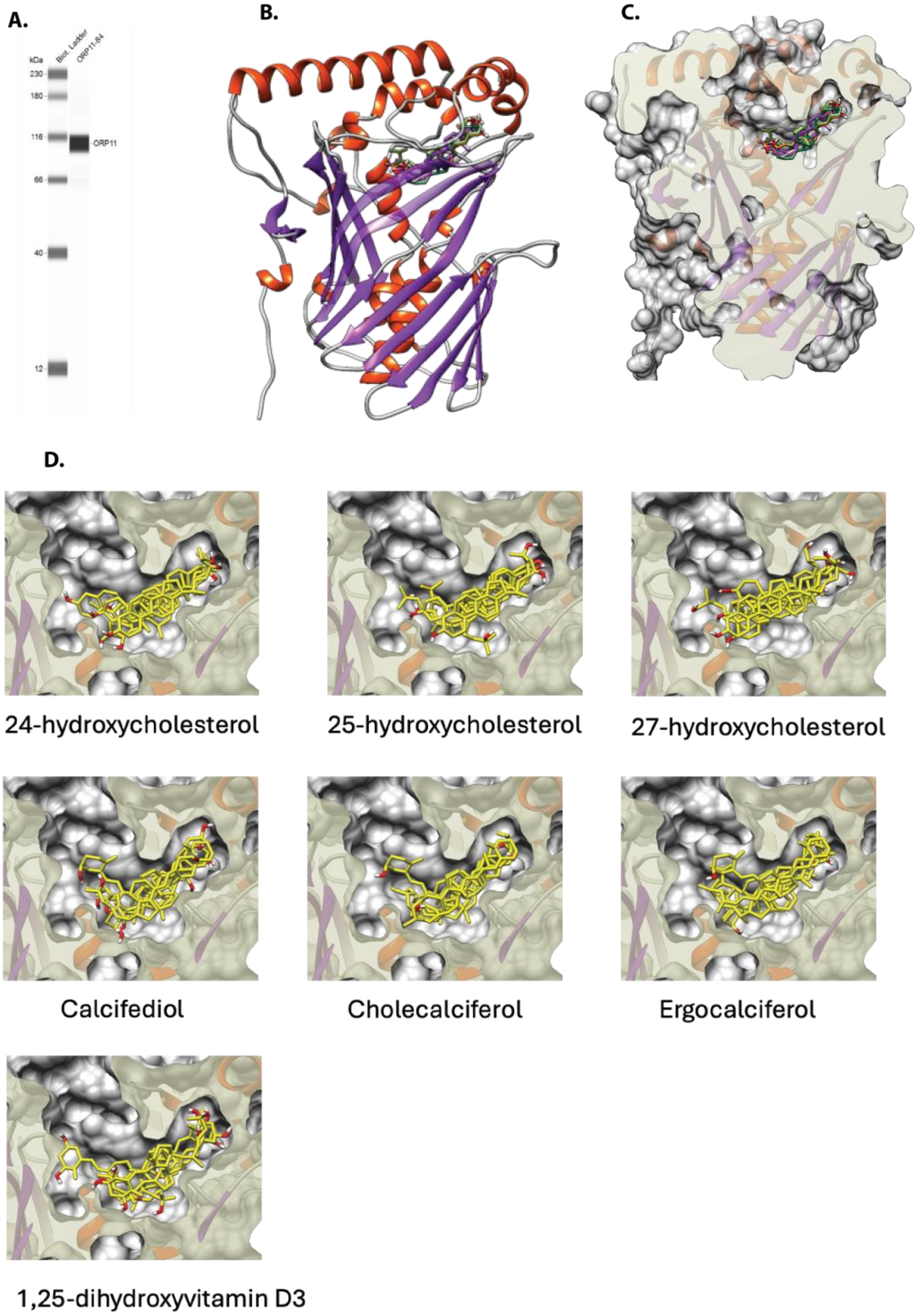

Supplementary Figure 7. OSBPL11 Alpha Fold model. Protein immunoassay of OSBPL11-myc-His overexpressed 293T lysate (A), OSBPL11 AlphaFold model (B), molecular docking of oxysterols and vitamin D compounds into oxysterol binding site in the AlphaFold modeled OSBPL11 (C), visualization of all binding poses for individual sterols and vitamin D analogs (D). Docking was performed using AutoDock Vina (v. 4.2.6, see Web Resources) plugin through UCSF Chimera (v. 1.17.3). Figures were made in UCSF Chimera (see Web Resources).

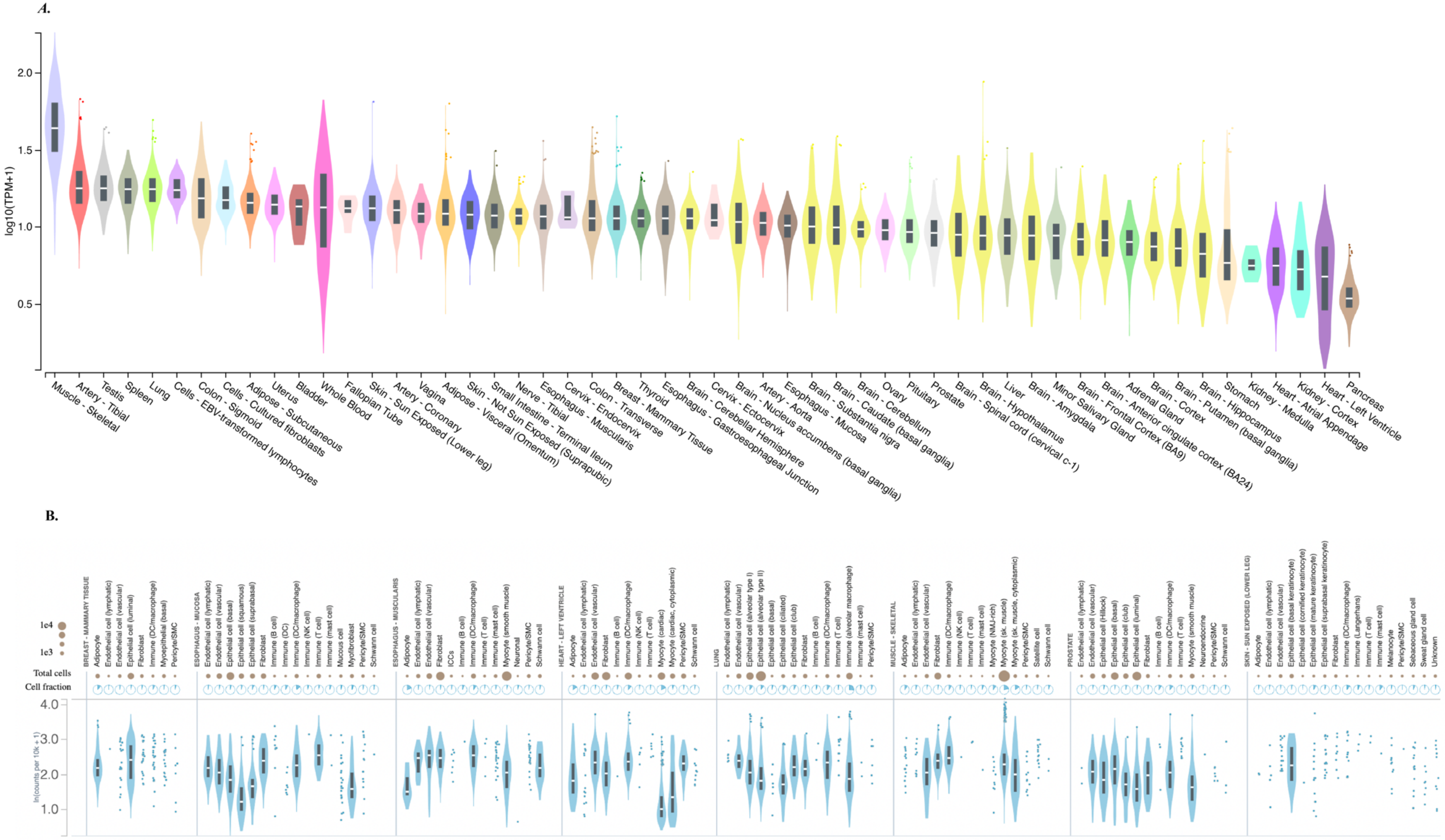

Supplementary Figure 8. Bulk tissue (A) and single-cell (B) gene expression for *OSBPL11* (ENSG00000144909.7). Gene expression data were obtained from GTEX Portal version V8 (https://gtexportal.org/).

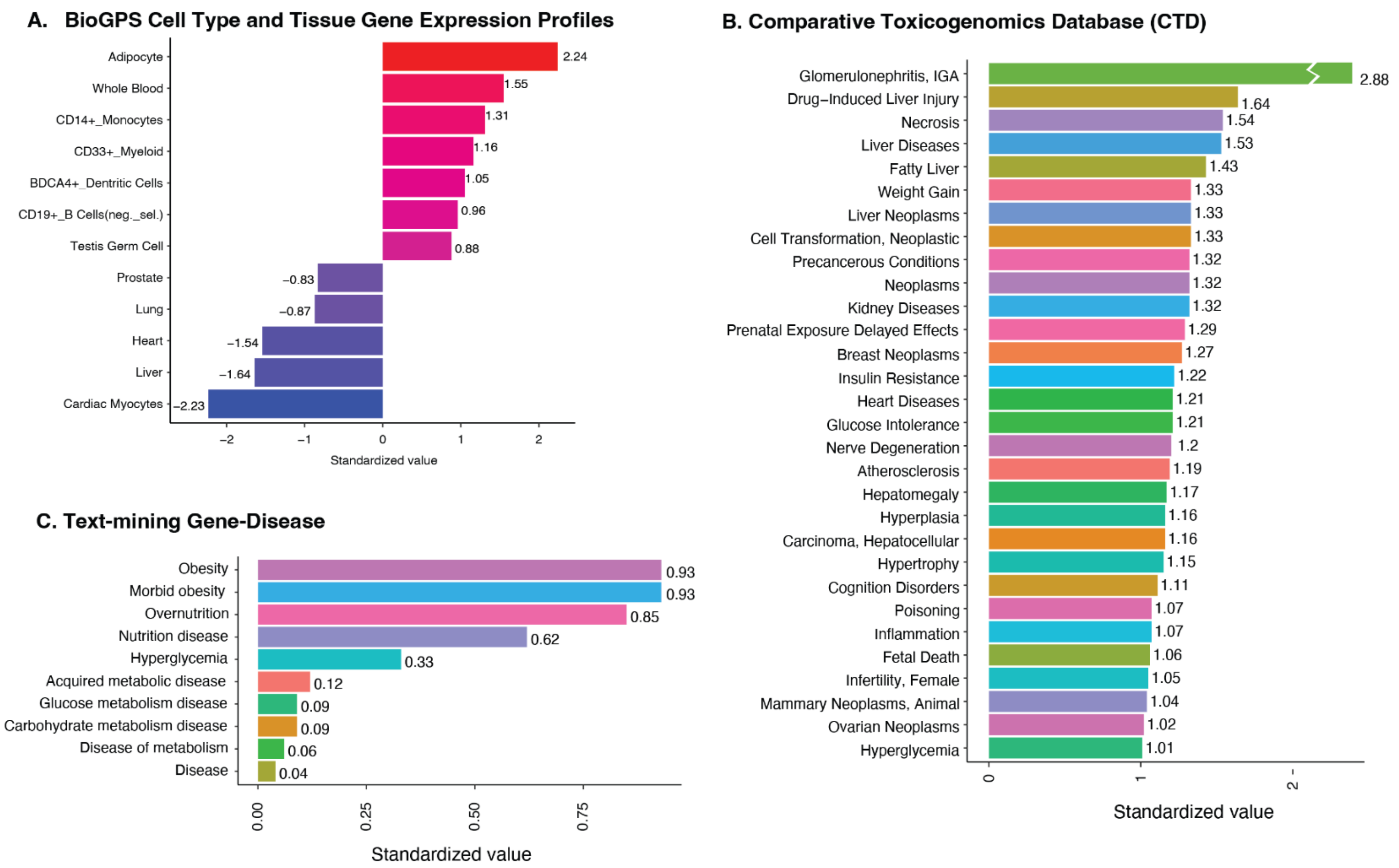

Supplementary Figure 9. Select *OSBPL11* search results from Harmonizome portal: differential gene-expression profiles in human tissues/cells (A) and mouse cell types in BioGPS datasets (B), GEO Signatures of Differentially Expressed Genes for Diseases (C), association of *OSBPL11* with diseases in Comparative Toxicogenomics Database (CTD) (D) and Text-mining Gene-Disease dataset (E). Complete Harmonizome (https://maayanlab.cloud/Harmonizome/) search results are presented in Supplementary Dataset 6. The standardized values shown represent z-scores calculated in the Harmonizome database, indicating how many standard deviations each gene's measurement is from the mean of the dataset, thus allowing for direct comparison across different datasets.

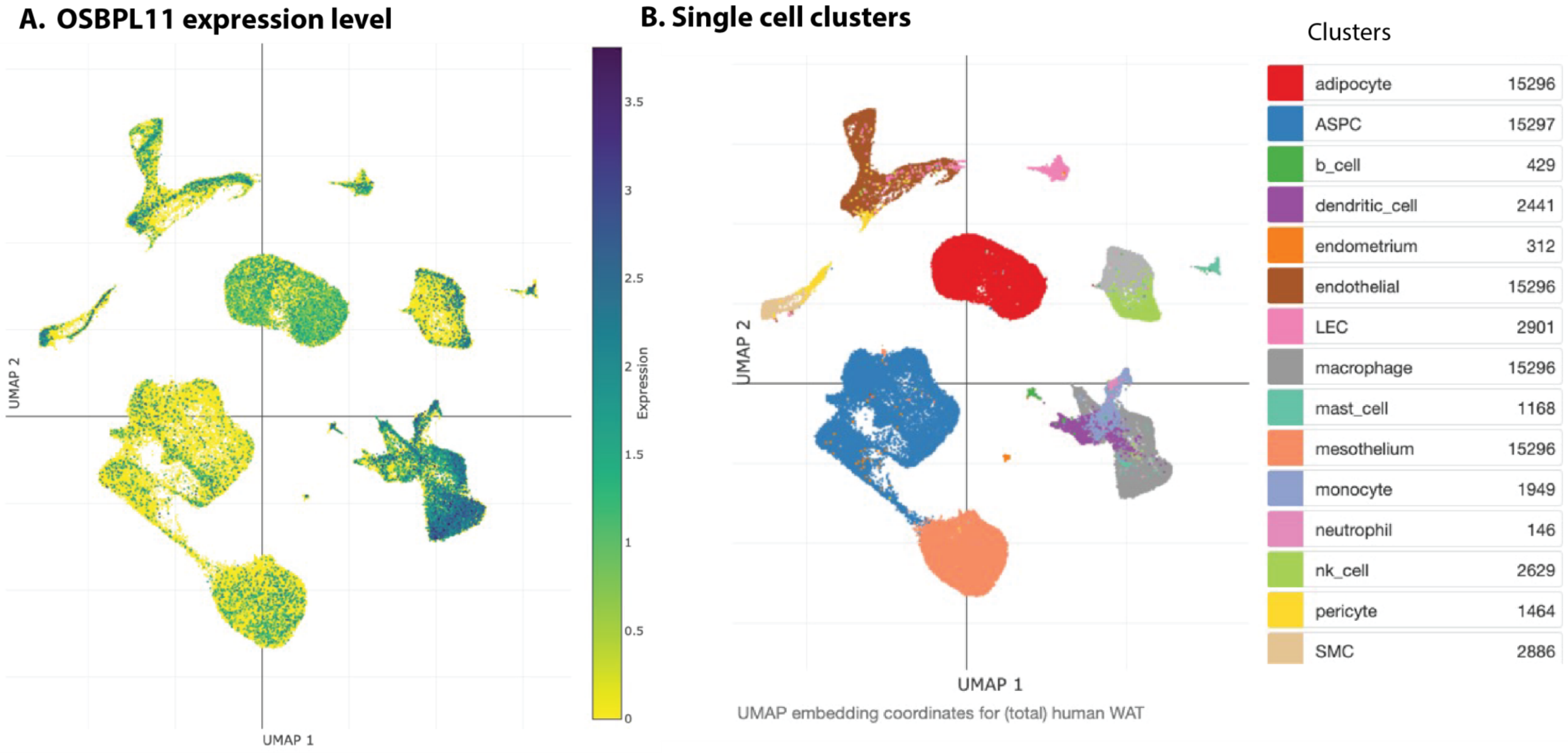

Supplementary Figure 10. Single cell atlas of human white adipose tissue showing expression levels for *OSBPL11*. Data was retrieved from https://singlecell.broadinstitute.org/single_cell/study/SCP1376/a-single-cell-atlas-of-human-and-mouse-white-adipose-tissue. Abbreviations: OSBPL11, oxysterol binding protein like 11; UMAP, Uniform Manifold Approximation and Projection; B cell, B lymphocyte; T cell, T lymphocyte; LEC, lymphatic endothelial cell; NK cell, natural killer cell; SMC, smooth muscle cell; ASPC, adipose stem and progenitor cell.

medRxiv preprint doi: https://doi.org/10.1101/2025.05.27.25328359; this version posted May 31, 2025. The copyright holder for this preprint
(which was not certified by peer review) is the author/funder, who has granted medRxiv a license to display the preprint in perpetuity.
It is made available under a CC-BY 4.0 International license .

A. Signficant phenotypes

Phenotype	System	Allele	Zygosity	Life stage	Significant sexes	Significant P-value
**decreased circulating triglyceride level**		*Osbpl11*$^{tm1b(EUCOMM)Hmgu}$	Heterozygote	Early adult	♂	**1.66x10^{-5}**
**improved glucose tolerance**		*Osbpl11*$^{tm1b(EUCOMM)Hmgu}$	Heterozygote	Early adult	♂	**6.21x10^{-5}**
**increased total body fat amount**		*Osbpl11*$^{tm1b(EUCOMM)Hmgu}$	Heterozygote	Early adult	♂	**2.66x10^{-6}**
**preweaning lethality, complete penetrance**		*Osbpl11*$^{tm1b(EUCOMM)Hmgu}$	Homozygote	Early adult	⚥	**N/A ***

B. Physiological systems

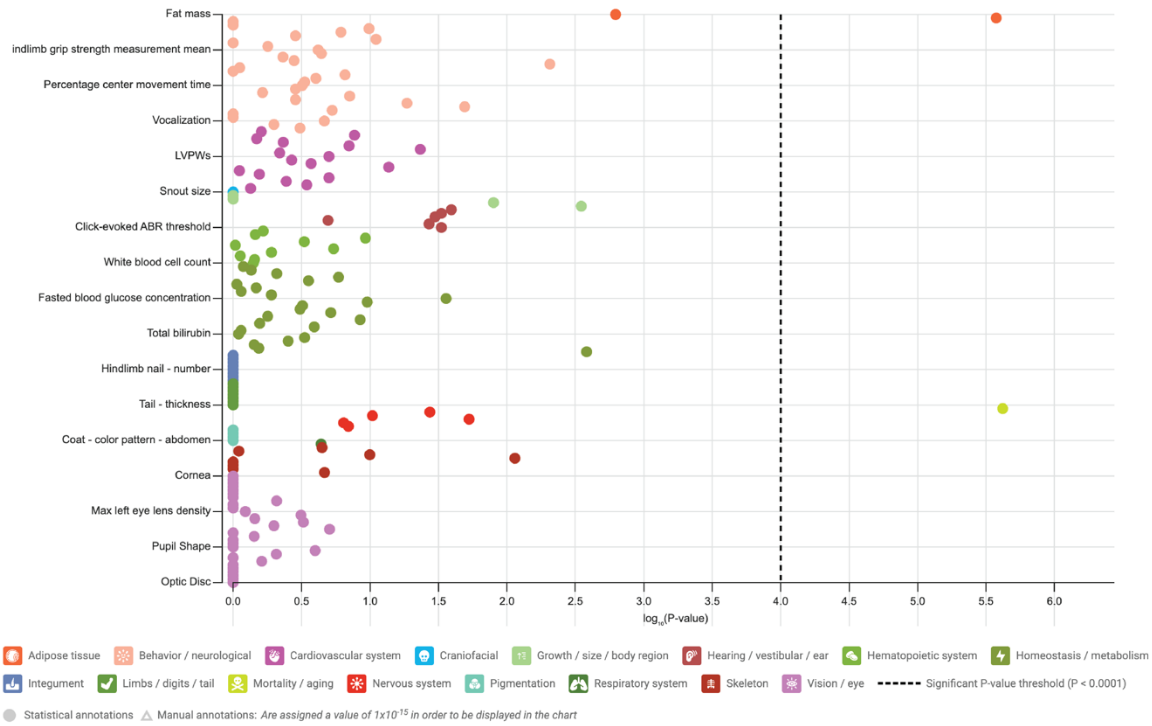

Supplementary Figure 11. Phenotypes with significant changes (A) and phenotyping screening results of Osbpl11 mouse knockout mice (B). The International Mouse Phenotyping Consortium (IMPC) applies a panel of phenotyping screens to characterize single-gene knockout mice in comparison to wild types. Data sourced from the IMPC (https://www.mousephenotype.org/data/genes/MGI:2146553).
